# Resistance training and clinical status in patients with postdischarge symptoms after COVID-19: protocol for a randomized controlled crossover trial “The EXER-COVID Crossover Study”

**DOI:** 10.1186/s13063-022-06608-y

**Published:** 2022-08-09

**Authors:** Robinson Ramírez-Vélez, Julio Oteiza, Juan Manuel Casas Fernández de Tejerina, Nora García-Alonso, Gaizka Legarra-Gorgoñon, Sergio Oscoz-Ochandorena, Hugo Arasanz, Yesenia García-Alonso, María Correa-Rodríguez, Mikel Izquierdo

**Affiliations:** 1 Navarrabiomed, Hospital Universitario de Navarra (HUN), Universidad Pública de Navarra (UPNA), Instituto de Investigación Sanitaria de Navarra (IdiSNA), Pamplona, Spain; 2 CIBER of Frailty and Healthy Aging (CIBERFES), Instituto de Salud Carlos III, Madrid, Spain; 3 Servicio de Medicina Interna, Hospital Universitario de Navarra (HUN), Universidad Pública de Navarra (UPNA), Instituto de Investigación Sanitaria de Navarra (IdiSNA), Pamplona, Spain; 4 Oncoimmunology Group, Navarrabiomed, Instituto de Investigación Sanitaria de Navarra (IdiSNA), Pamplona, Spain; 5 Medical Oncology Department, Hospital Universitario de Navarra (HUN), Universidad Pública de Navarra (UPNA), Instituto de Investigación Sanitaria de Navarra (IdiSNA), Pamplona, Spain; 6 Department of Nursing, Faculty of Health Sciences, University of Granada, 18016 Granada, Spain; 7 Biosanitary Research Institute (ibs.GRANADA), Granada, Spain

**Keywords:** COVID-19, Multicomponent exercise, Inflammation, Immunology

## Abstract

**Background:**

Physical exercise induces a coordinated response of multiple organ systems, including the immune system. In fact, it has been proposed that physical exercise may modulate the immune system. However, the potential effect of an exercise program on COVID-19 survivors has not been investigated. Thus, the aim of this study is to evaluate the modifications in immunological parameters, physical condition, inflammatory profile, and perceived persistent symptoms after 6 weeks of supervised resistance training (RT), in addition to the standard care on the clinical status of patients with persistent COVID-19 symptoms. The objective of this protocol is to describe the scientific rationale in detail and to provide information about the study procedures.

**Methods/design:**

A total of 100 patients with postdischarge symptoms after COVID-19 will be randomly allocated into either a group receiving standard care (control group) or a group performing a multicomponent exercise program two times a week over a period of 6 weeks. The main hypothesis is that a 6-week multicomponent exercise program (EXER-COVID Crossover Study) will improve the immunological and inflammatory profile, physical condition, and persistent perceived symptoms (fatigue/tiredness, musculoskeletal pain, and shortness of breath) in patients with postdischarge symptoms after COVID-19.

**Discussion:**

Our results will provide insights into the effects of a multicomponent exercise program on immunological parameters, physical condition, inflammatory profile, and persistent perceived symptoms in patients with postdischarge symptoms after COVID-19. Information obtained by this study will inform future guidelines on the exercise training rehabilitation of patients with postdischarge symptoms after COVID-19.

**Trial registration:**

NCT04797871, Version 2. Registered on March 15, 2021.

**Supplementary Information:**

The online version contains supplementary material available at 10.1186/s13063-022-06608-y.

## Background and rationale

In 2019, a new coronavirus was identified from an outbreak originating in the city of Wuhan, China. At present, this virus is known as severe acute respiratory syndrome coronavirus 2 (SARS-CoV-2), and the resulting disease is known as coronavirus disease 2019 (COVID-19). On January 30, 2020, the World Health Organization (WHO) declared this disease a public health emergency of international scope, and in less than 2 months, on March 11, it was classified as a pandemic [1]. The clinical picture associated with this virus is referred to as COVID-19. In cases of COVID-19, the SARS-CoV-2 virus promotes an inflammatory response in the lung parenchyma that is characteristic of the pathology of SARS and is mediated by the cytokine interleukin-1β (IL), which is activated by the inflammasome complex IL-6 and tumoral necrosis factor-alpha (TNF).

A characteristic of the condition produced by this pathogen is that when symptoms appear, the condition can worsen very quickly, although virulence is related to pathogen/host symbiosis. The primary symptoms are fever (80%), nonproductive cough (56%), fatigue (22%), and myalgia (7%), and these may coexist with symptoms related to a possible neurotropism, such as anosmia or headache [2]. Other symptoms may include dyspnea, chills, odynophagia, headache, angina, or conjunctivitis [3]. Although COVID-19 symptoms can be mild or moderate for most people, the disease can lead to serious medical complications and, in some cases, death [3]. Additionally, older adults and people with chronic noncommunicable comorbidities, such as diseases of the cardio-cerebro-circulatory system, certain types of cancer and pulmonary, metabolic, and/or renal diseases, have a higher risk of becoming ill with COVID-19 and developing serious complications.

Because COVID-19 is a new disease, much of its clinical course remains uncertain, particularly the possible long-term health consequences, if any [4]. For example, it is estimated that approximately 20 million people worldwide have completely recovered from COVID-19; however, patients with persistent severe symptoms and even substantial target organ dysfunction have been reported after SARS-CoV-2 infection [5]. Along this line, the COVID-19 Symptomatology Study reports that 1 in 10 patients diagnosed with SARS-CoV-2 remain symptomatic beyond 3 weeks, and some of them have persistent symptoms 6 months after the acute picture [6]. Although there is no consensus definition of chronic or persistent COVID-19 (understood as symptoms that extend beyond 12 weeks), it has been reported that COVID-19 survivors frequently report symptoms such as chronic fatigue, dyspnea on minimal exertion, joint pain, and/or chest pain [7]. In addition to these symptoms, coexisting dysfunction of specific tissues, such as the heart, lung, and brain, has been reported [8].

Although the etiopathogenesis of the symptomatology in patients with chronic COVID-19 is not clear, it has been hypothesized that these complications could be the consequence of direct tissue invasion by the SARS-CoV-2virus, possibly mediated by (*i*) the presence of angiotensin-converting enzyme receptor 2 (ACE-2), (*ii*) the exacerbated inflammatory response (also called cytokine storm), which is associated with damage to the immune system, or (*iii*) the state of hypercoagulability described in severe COVID-19.

In terms of persistent symptoms (for example, pain and/or chronic fatigue), several studies have described a relationship between the activation of the cannabinoid system and regular physical exercise (especially at high training intensities), which would partially explain effects of exercise on improving mood, pain perception, and fatigue in humans [9, 10]. In animal studies, authors of [11, 12] have described the analgesic activity resulting from the regulation of activation of the endocannabinoid system, and authors such as Crombie et al. [13] reported that a single session of moderate- to vigorous-intensity physical exercise was sufficient to induce analgesia through endocannabinoids and described the effect of such activity on the CB1 and CB2 receptors at the peripheral, medullary, and supramedullary levels in rats, suggesting plausible molecular mechanisms that could be applied to patients with chronic pain and/or fatigue conditions, such as those reported in patients with COVID-19. The excessive consumption of alcohol also increases the risk of contracting diseases associated with severe manifestations of COVID-19 [14], not because of deterioration in the physiological function of target organs and general immune function but also because of the negative effects of the COVID-19 pandemic on health and social behavior. Overweight and obesity are among the most common comorbidities in patients hospitalized with COVID-19, in part due to the low-grade subclinical inflammation associated with excess weight/adiposity, which deteriorates the immune response to viral infection, and/or to the close relationship between type 2 diabetes and a state of oxidative stress that limits cardiovascular and respiratory function [15]. In addition, physical inactivity can indirectly influence the progression and complications of COVID-19 [16]. This is because physical inactivity is associated with obesity/overweight, and metabolic risk factors are associated with a severe picture of COVID-19. Consequently, it is important to maintain a healthy lifestyle, especially the practice of physical exercise.

### Exercise can improve clinical status in patients with postdischarge symptoms after COVID-19

There is irrefutable evidence of the beneficial role of physical exercise in the prevention of disease, as a complementary treatment for chronic diseases and in promoting psychological well-being [16–18]. In addition to promoting optimal physical and mental health, an active and healthy lifestyle throughout the lifespan strengthens and improves immune function and even reduces the risk of viral and/or chronic degenerative diseases [17]. Exercise may have a protective effect on the immune system, whose optimal state is crucial to an appropriate response to the threat of COVID-19 [18].

The mechanisms by which physical activity influences the immune system have been little elucidated and are currently a subject of debate, but it is possible to affirm certain physiological and psychological perceptions, which are described as follows: Physical exercise, when practiced regularly, increases the subject’s immune competence (that is, the ability of lymphoid cells to produce a humoral or cellular immune response when stimulated by an antigen) [19]. This benefit seems to be accentuated among older adults, reducing their immunosenescence and delaying immune aging [20]. This increase in immune competence occurs through increased concentrations of CD4+ cells, which are responsible for detecting pathogens and activating the immune response [21].

Physical exercise, through different mechanisms related to the sympathetic, humoral, and hormonal response, favors the release of myokines from skeletal muscle, which can help maintain immune competence. It has been shown that muscle-derived interleukin- (IL-) 6 directs immune cell trafficking to areas of infection, while IL-7 can stimulate the production of thymus T cells, and IL-15 helps maintain T cell and natural killer (NK) cell homeostasis in the periphery. Some myokines also have antiproliferative effects that can induce apoptosis (programmed cell death) in solid tumor cells. All these cells work together to increase immune defense [22].

Physically active people show better control of latent viral infections, even during periods of stress such as isolation and confinement, events that can inhibit many critical functions of the immune system through the increase in glucocorticoids (for example, cortisol) [23]. Therefore, the ability of physical activity to mitigate the harmful effects of stress on immunity is highlighted. Physical activity seems to strengthen the immune response of the mucous membranes by increasing the concentration of immunoglobulin IgA, which could be beneficial for preventing some types of infections, particularly respiratory infections such as COVID-19 [24]. In older adults, an amplification of the T helper 1 (TH1) lymphocyte pattern without changes to TH2 has been described. That is, regular physical activity increases the number of T lymphocytes, B cells, and NK cells and increases the production of interferon gamma and IL-6 [25]. It has also been reported that physical activity can stimulate immune function and reduce inflammation and has several physiological and psychological benefits, such as the reduction of stress and anxiety [26]. Exercise also significantly increases antibody levels after vaccination, especially in older adults; therefore, the results of advances in this field should focus primarily on vulnerable people, such as health professionals, elderly adults and patients with neoplasms, chronic diseases, and immunosuppression [27].

### Clinical outcomes responses to resistance training are unknown in patients with postdischarge symptoms after COVID-19

Resistance training (RT) has modulating effects in humans, a finding that is of great importance for the immune system’s response to various infectious agents in cardiovascular degenerative conditions and in cases of chronic immunodeficiency [28–30]. It has been reported that the effect of RT changes according to its intensity, the presence of chronic immunodeficiency and age [31–33]. In addition, RT, but not intense exercise, has a protective effect [34–36]. Markers of endothelial inflammation and stress and lymphocyte subpopulation (LSP) change with RT, according to the current literature [28, 37]. This effect has been described in humans who engage in acute exercise at different intensities, but the effect of chronic RT on COVID-19 survivors has not been determined.

Considering that in postdischarge symptoms after COVID-19 population, the exercises that are achieved may be psychologically demanding, due to heightened cardiovascular and neuromotor effort, it is surprising that an examination of clinical status outcomes in RT for the post-COVID-19 syndrome population is not currently available. An understanding of cardiovascular and immunological parameters of response to exercise, like psychosocial outcomes, and how RT may affect such outcomes, would provide important information about the potential sustainability of exercise and its applicability in post-COVID-19 syndrome rehabilitation. To the best of our knowledge, we proposed a first clinical trial of randomized controlled crossover design using international recommendations with an emphasis on twice-a-week strength training, in patients with persistent COVID-19 symptoms [38].

### Objectives

The primary objective of this study is to compare the effects of 6 weeks of RT vs. standard care on the clinical status of patients with persistent COVID-19 symptoms. Changes in clinical status will be determined by examining changes in perceived persistent symptoms (i.e., fatigue/tiredness, musculoskeletal pain, shortness of breath). In addition, the secondary objective of this study is to compare the effects of RT on measures of immunological and inflammatory markers, cardiovascular health, and psychosocial responses to exercise. Changes in immunological and inflammatory markers will be measured by assessing lymphocyte immunophenotypes and cytokine assays. Changes in cardiovascular health will be measured by assessing resting blood pressure, arterial stiffness, heart rate variability, maximal aerobic capacity (V̇O_2_max), muscle strength, and body composition. Psychosocial responses to exercise will examine the effect of RT on perceived persistent symptoms (i.e., fatigue/tiredness, musculoskeletal pain, shortness of breath) in response to the exercise intervention. The RT exercise intervention will be conducted using a whole-body paradigm, which will utilize Smart Strength machines (eGym® GmbH, München, Germany) and cycle ergometer.

### Research hypothesis

We hypothesize that RT will be more effective than standard care in improving clinical health status. The main hypothesis is that by applying a supervised RT (EXER-COVID Crossover Study) for 6 weeks, we could reduce the perception of persistent symptoms. We could increase physical performance (V̇O_2_max and/or muscle fitness) through the effects of mechanical and endocrine action, and we could modulate the production of immunological and inflammatory markers due to skeletal muscle contraction, which could contribute to reducing the inflammatory process that is usually established in COVID-19 infection. Exercise training is also expected to be more effective in improving cardiovascular measures of resting blood pressure, arterial stiffness, and heart rate variability as compared to no training. If successful, our trial would provide evidence that exercise training has potential for patients with postdischarge symptoms after COVID-19.

## Methods

### Study design

The trial will be a monocentric, randomized, crossover design registered with the Clinical Trials Registry (NCT04797871 [First Posted: March 15, 2021, Last Update Posted: May 17, 2022]). All items from the ClinicalTrials.gov registry platform can be found within the protocol. This is a superiority trial, with equal allocation to intervention and control groups. The protocol will be developed according to the Standard Protocol Items: Recommendations for Interventional Trials (SPIRIT) guidelines (see Additional file 1) for randomized controlled trials (RCTs) [39].

### Participant eligibility

A total of 100 adults of both sexes between 20 and 60 years old will be recruited from the Hospital Universitario de Navarra (HUN). At the HUN site, participants will be admitted from internal medicine service referrals and from participant databanks of patients who have consented to be contacted for participation in research projects. All patients will be screened for inclusion by a physician to ensure that the patient has been diagnosed with COVID-19 and has no other psychiatric or somatic condition that can explain the persistent COVID-19 symptoms. Potential participants will fulfill the following inclusion criteria: over 18 years of age, diagnosed with SARS-CoV-2 using real-time reverse transcriptase polymerase chain reaction (PCR) tests or testing positive for SARS-CoV-2 virus antigen >90 days before randomization, presenting with a chronic symptomatic phase lasting >90 days since the onset of symptoms, has not been hospitalized, has no evidence on clinical records of pneumonia or any other organ failure related to SARS-CoV-2, and is capable and willing to provide informed consent. Patients previously treated for persistent COVID-19 symptoms (i.e., physical therapy or rehabilitation); patients who are pregnant or breast-feeding; patients with cardiovascular or endocrine comorbidities (i.e., atrial fibrillation, acute myocarditis, acute heart attack or unstable angina, aortic stenosis, acute endocarditis/pericarditis, uncontrolled high blood pressure, acute thromboembolism, severe heart failure, respiratory failure, and uncontrolled acute decompensated diabetes mellitus or low blood sugar) or other neurological or musculoskeletal comorbidities that will prevent safe exercise participation; patients with pain that is made worse with exercise; patients with cognitive, communication, or behavioral issues that may limit their safe participation in the exercise program, or contraindications that will prevent their participation in the exercise intervention; and patients who have been treated with IL-6 receptor antagonists (tocilizumab, kevzara) within the last month, due to drug interference with the cardiopulmonary exercise adaptations, will be excluded.

### Randomization, allocation concealment, and blinding

Eligible participants will be randomly assigned to one of 2 sequence orders with a 1:1 allocation using a computer-generated block randomization schedule (Research Randomizer V.4). Assignment will take place through previously constructed balanced blocks, which will allow equiprobable assignment and ensure that each group has the same number of participants. This allocation mechanism will be carried out until 100 total participants are assigned. The assignment keys will be kept in a confidential file in the Navarrabiomed center and will be opened at the end of the study’s analyses. To ensure masking, an alpha-numerical code will be assigned to each study group. This code will be delivered to the associated researcher and will not be revealed to the investigators in charge of processing the data until the analysis of the coded interventions is completed. Additionally, the researchers from the Navarrabiomed center will be responsible for preparing reports for the participants’ safety committee to verify the progress of the study. Following the conclusion of the baseline examination, the appropriate envelope will be opened by a study researcher, and the participants will be informed about their group allocation. Assessors of outcomes will be blinded to patient data, including allocation at baseline and follow-up. Blinding will be used for all laboratory analyses. Owing to the nature of the study, patients cannot be blinded to the exercise training modality.

### Procedure for unblinding if needed

Not applicable. This study is an unblinded, practice-level intervention.

### Procedure

**Fig. 1:**
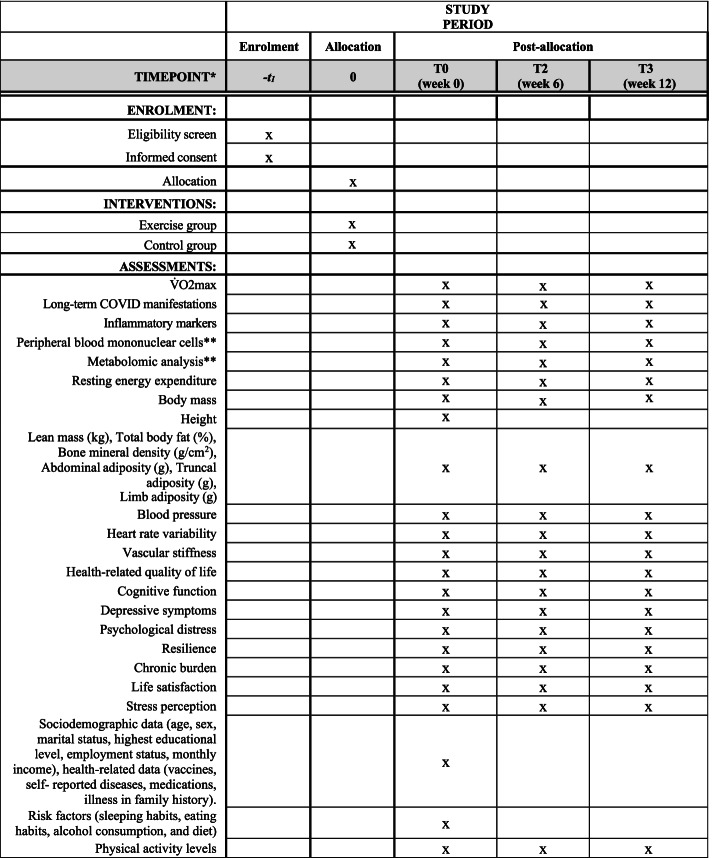
Schedule illustrating enrolment and interventions (SPIRIT figure) of the “EXER-COVID Crossover Study”. T: time-point; *According to SPIRIT 2013 statement. ** A random subsample of participants (20 subjects in each group, *n*=40)

**Fig. 2:**
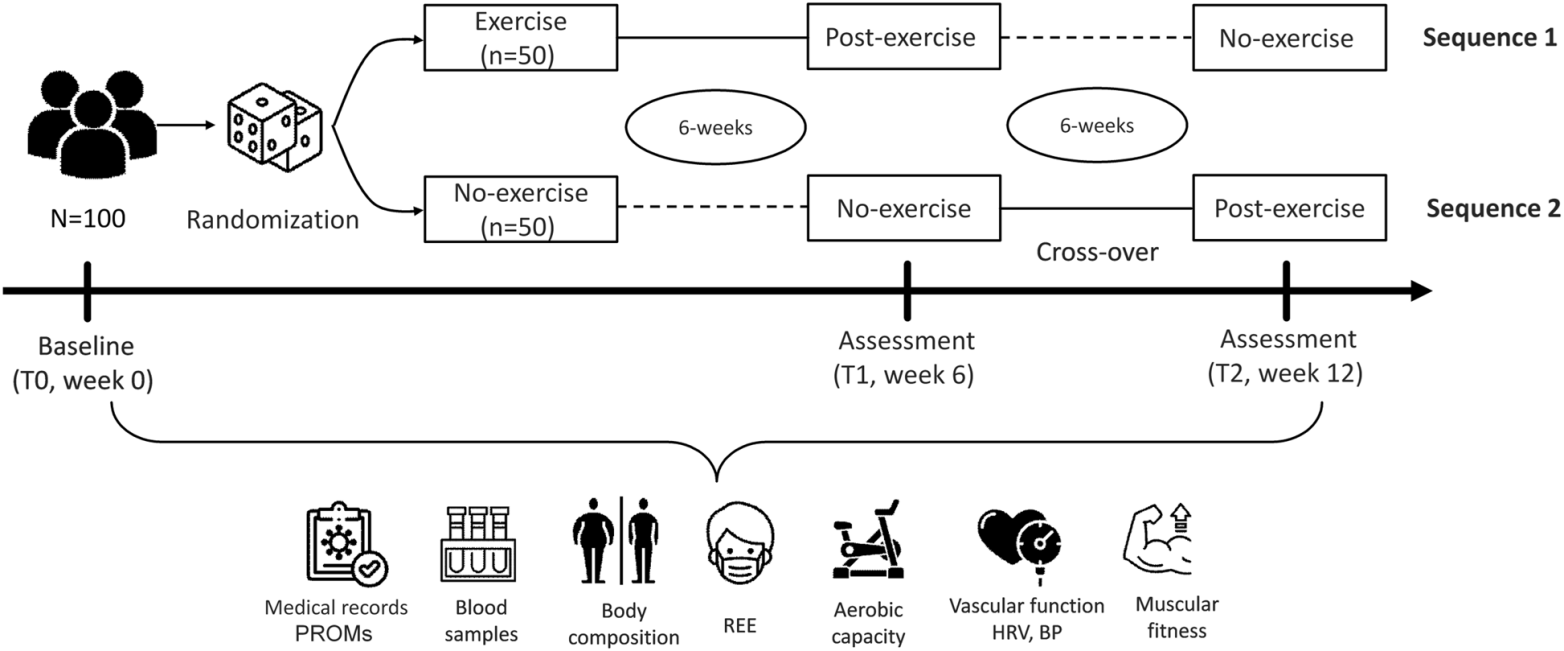
Flow of subjects through the “EXER-COVID Crossover Study”. Participants will be first randomly assigned to a 6-week period of no-exercise training (Sequence 1, *n*=50) or exercise training (Sequence 2, *n*=50) after which full crossover occurred. T: time-point; PROMs: patient-reported outcome measures; REE: resting energy expenditure; HRV: heart rate variability; BP: blood pressure

Participants will be randomly assigned (1:1) to one of 2 sequence orders using computer-generated random numbers. All participants will complete both group interventions. Therefore, as participants will act as their own control, this approach will remove any potential differences between subjects and reduce the potential bias when estimating the intervention effect. First, eligible and consenting participants will start with the baseline assessment (T0). Second, participants will be assigned to start 6 weeks of either control group (Sequence 1) or exercise training (Sequence 2), T1 (week 6). Third, full crossover occurred after the 6 weeks, during which participants completed a second 6-week period of the opposite condition (T2, week 12). Outcome assessments will be performed at the end of the immediate intervention and control phase and at the end of the crossover intervention phase (Fig. 1). The general procedure is shown in Fig. 2.

### Intervention

All interventions will take place at the Biomedical Research Center of Navarra (Navarrabiomed) site facilities. Standard medical care will be provided to all participants in all groups. The exercise intervention will consist of 6 weeks of individualized supervised muscle power RT, two sessions per week performed on nonconsecutive days to avoid overtraining and maximize adaptations. To reduce the risk of COVID-19 reinfection, a 1:1 trainer-to-participant format will be used during training sessions. On the training days, the participants will warm up with 3 sets of 10 repetitions of half air squats with the participants’ own body weight followed by 10 min on an electronically braked cycle ergometer (Lode Excalibur Sport, Groningen, The Netherlands) at 50% of the VO_2_peak and 4 sets of 30 s followed by 90 s of passive recovery at 100–120% of the VO_2_peak. EGYM Smart Strength machines (eGym® GmbH, München, Germany) will be used for both RT and maximum strength and muscle power measurements of the lower and upper extremity muscles. Muscle power RT, including motivational gamification and maximum acceleration of constant weight from 50 to 70% of daily maximum strength measurements, will be used during training (Explonic eGym® intelligent training program).

The RT intervention will comprise 3 sets of 8 to 12 repetitions with a load equivalent to 50 to 70% of the daily 1-repetition maximum (1 RM) for all major muscle groups (i.e., the exercises for the upper body include pectoral press and back press, and the exercises for the lower body include leg press and leg extensions). All movements will be performed at 100% of the maximum speed achieved during the propulsive phase (i.e., as fast as possible according to the patient’s muscular and functional capacity). The recovery between sets will be between 1 and 2 min. At the end of the session, there will be 5 min of stretching to cool down. The duration of the sessions will be 30–45 min, and the duration of the entire program will be 6 weeks, equivalent to 12 sessions. See Table 1 for details.

**Table 1:** Supervised exercise program for “EXER-COVID Crossover Study”

Weeks	1		2		3		4		5		6	
***Session***	***1***	***2***	***3***	***4***	***5***	***6***	***7***	***8***	***9***	***10***	***11***	***12***
Warm up	3 sets of 10 reps of bodyweight half squat+ 10 min at cycling at 50% of V̇O_2_peak+4 sets of 30 s at 100–120% of V̇O_2_peak											
Strength training												
Exercises	Pectoral press, back press, squat press and knee extension											
Warm up	2 sets of 5 reps at 30–40% of daily maximum strength											
Sets	3	3	3	3	3	3	3	3	3	3	3	3
Repetitions	10	10	12	12	10	10	12	12	8	8	10	10
Intensity (1 RM %)	50	50	50	50	60	60	60	60	70	70	70	70
Cool down												
Stretching	5 min of static and dynamic flexibility exercises											

*1 RM* one-repetition maximum

The no-exercise (control group) will receive standard hospital care, which includes rehabilitation when necessary. Standard care will rigorously conform with all the standard care steps in the current COVID-19 management protocol, and participation will not affect the patients’ treatment or the care they receive during recovery from the disease during the study or in the future. As described at the beginning of the procedure section, this protocol will use a crossover design in which the intervention group will receive the intervention (RT) during the first phase (6 weeks) followed by standard medical care during the second phase (6 weeks). The control group will receive the treatments in the reverse order (standard treatment first followed by the RT intervention). This will allow us to make individualized adjustments, allowing each subject to serve as their own control. If participants desire to stop training and develop health conditions (i.e., SARS-CoV-2 reinfection) or injury that precludes safe participation of exercise over the course of the intervention, we will discontinue training. Participants will be asked to not participate in another structured exercise regimen or intervention over the course of their participation in either the intervention or control group; otherwise, they will not be allowed to continue participation.

### Outcome measures

The measurements include a medical health interview and examination (e.g., body measures, blood pressure, heart rate, ECG), a V̇O_2_max test, a whole-body dual-energy X-ray absorptiometry (DXA) scan, blood samples, 1-repetition max for upper and lower body muscles, and questionnaires (PROMs: patient-reported outcome measures, physical activity levels, diet recall, quality of life, etc.). The questionnaires will be administered online using SurveyMonkey. Participants will receive an email with a personal link to fill out the questionnaires. For optimal retention, participants will be contacted by the research assistant if they have not completed the questionnaires within 1 week. Outcome measures will be assessed at T0, T1, and T2. See Fig. 2 for the measurements at all time points.

### Primary outcome

The primary outcome is the change in aerobic exercise capacity measured by (V̇O_2_max) before and after the intervention period. Graded exercise tests will be performed on a bicycle ergometer (Lode B.V., Groningen, The Netherlands) to determine the maximal oxygen uptake. The test will start with a 3-min warm-up at 25 W. Warm-up will be immediately followed by a 25-W increase every 2 min until exhaustion, which is categorized as voluntary exhaustion or failure to maintain rounds per minute above 60. Breath-by-breath gas exchange data will be collected using a metabolic gas analyzer (QUARK, Cosmed, Rome, Italy) and used to determine V̇O_2_max and total energy expenditure during exercise. The staggered phase will have a maximum duration of 8 to 12 min, and the cadence of the pedal will not be altered during the effort (approx. 1.23 Hz or 50–60 RPM). The heart rate (HR) will be measured with a cardiotachometer (Polar Vantage NV, Finland) every 5 s.

### Secondary outcomes

#### Long-term COVID manifestations

Data on specific symptoms potentially correlated with long-term COVID-19 will be obtained using a standardized questionnaire administered at enrollment via PROMs [40]. At baseline and at the end of each crossover period, participants will be asked about the presence or absence of symptoms related to long-term COVID-19 and whether each symptom persisted at the time of the visit. More than 1 symptom could be reported that reflects symptoms such as functional limitations and clinical characteristics (i.e., nausea/vomiting, impaired visual acuity or blurry vision, anosmia, dizziness, depression, chills, weakness, musculoskeletal pain, palpitations/tachycardia, change of appetite, frustration, cognitive involvement, anxiety/irritability, dryness, impaired concentration, and headache).

#### Inflammatory markers

Inflammatory markers will be assessed following an overnight fast (10–12 h). Blood samples (2.7 ml EDTA plasma, 7.5 ml serum, 4.9 ml lithium-heparin, 5 ml citrate plasma) will be collected and processed by a trained laboratory nurse and analyzed according to standard procedures. Plasma will be stored at −80 °C prior to analysis. Blood samples will be analyzed for preliminary medical examination (i.e., full blood count, albumin, creatinine, urea, potassium, sodium, calcium, CRP, GGT, ALT, D-dimer, creatine kinase, TSH, T3, and T4). At the two experimental study visits (T0–T1 and T2), blood (2 × 7.5 ml serum) will be collected for a cytokine human immune monitoring panel using a commercial array (ProcartaPlex™, MAGPIX 65-Plex, Ref: EPX650-10065-901), including *cytokines* G-CSF (CSF-3), GM-CSF, IFN alpha, IFN gamma, IL-1 alpha, IL-1 beta, IL-2, IL-3, IL-4, IL-5, IL-6, IL-7, IL-8 (CXCL8), IL-9, IL-10, IL-12p70, IL-13, IL-15, IL-16, IL-17A (CTLA-8), IL-18, IL-20, IL-21, IL-22, IL-23, IL-27, IL-31, LIF, M-CSF, MIF, TNF alpha, TNF beta, and TSLP; *chemokines* BLC (CXCL13), ENA-78 (CXCL5), Eotaxin (CCL11), Eotaxin-2 (CCL24), Eotaxin-3 (CCL26), Fractalkine (CX3CL1), Gro-alpha (CXCL1), IP-10 (CXCL10), I-TAC (CXCL11), MCP-1 (CCL2), MCP-2 (CCL8), MCP-3 (CCL7), MDC (CCL22), MIG (CXCL9), MIP-1 alpha (CCL3), MIP-1 beta (CCL4), MIP-3 alpha (CCL20), and SDF-1 alpha (CXCL12); *growth factors/regulators* FGF-2, HGF, MMP-1, NGF beta, SCF, and VEGF-A; and *soluble receptors* APRIL, BAFF, CD30, CD40 L (CD154), IL-2R (CD25), TNF-RII, TRAIL (CD253), and TWEAK. Molecules showing significant differences between the two patient cohorts in marker levels and changes will be validated by ELISA. Blood samples will be stored at − 80 °C for future potential analyses, such as metabolomics, exosome isolation, and/or molecular analyses.

### Isolating the peripheral blood mononuclear cells (PBMCs) and metabolomic analysis

A random subsample of participants (20 subjects in each group, *n*=40) will be included to isolate the PBMCs by Ficoll gradient, as previously described [41]. The following fluorochrome-conjugated antibodies will be used: CD4, CD8, PD1, CD62L, KLRG1, CD28, CD57, CD163, CD119, CD56, CD116, CD66b, CD11b, and CD14. Cells will be washed, stained and analyzed by flow cytometry. In addition, plasma lipid extraction will be performed and then analyzed by ultrahigh-resolution liquid chromatography coupled to mass spectrometry (UHPLC–MS) using ultrahigh-resolution liquid chromatography coupled to mass spectrometry (UHPLC–MS) using the technique described by Barr et al. [42]. In summary, plasma lipid extraction is a liquid extraction of the serum with chloroform/methanol (2:1) and a saline solution of sodium chloride followed by incubation the mixture for 1 h at −20 °C to promote the precipitation of proteins. Next, the sample is evaporated in an evaporating concentrator, and the dry extract is reconstituted in acetonitrile/isopropanol. After centrifugation, the supernatant is analyzed by UHPLC–MS. For this purpose, the chromatographic column UPLC BEH C18, 2. 1×100 mm, 1.7 μm (Waters Corp. Milford, MA, USA) will be used, with a mobile phase consisting of a binary mixture formed by H_2_O/acetonitrile/10 mM ammonium formate and acetonitrile/isopropanol/10 mM ammonium formate. The conditions of chromatographic separation and high-resolution mass spectrometry (Xevo G2 QToF, Waters Corp.) have been previously described [43].

### Resting energy expenditure

The resting energy expenditure (REE) will be measured using an automated metabolic gas analysis system (Q-NRG+ COSMED Rome, Italy). Participants will be instructed to lie in a supine position while enclosed in a clear hard plastic canopy, which is attached to the metabolic cart and dilution pump via a breathing tube. To do this, patients will lie down for 5–10 min. V̇O_2_ and carbon dioxide production (V̇CO_2_) will be measured for 30 min. Measurements will be taken first thing in the morning after fasting for 10 to 12 h, without smoking or consuming alcoholic beverages, and before performing any physical activity or personal hygiene. To avoid possible measurement biases and to decrease the variability between subjects, controls and calibrations of the CO_2_ and environmental thermoneutrality (temperature between 20 and 24 °C) will be carried out before each test, as indicated by the manufacturer. Additionally, weekly self-calibrations will be performed using a standard gas mixture of 95% O_2_ and 5% CO_2_. As recommended by the manufacturer, a nonprotein stoichiometric equation will be used to estimate the resting fat oxidation rate = (1.695 × V̇O_2_–1.701 × V̇CO_2_) [44].

### Anthropometrics and body composition

Participants will be tested wearing minimal clothing and barefoot. Height will be measured to the nearest 0.1 cm with a portable stadiometer (Seca 206, Seca Ltd, Birmingham, UK). Body mass will be determined to the nearest 0.1 kg using a calibrated scale (Seca HV120, Seca Ltd, Birmingham, UK) with the participant in light clothing. Body mass index will be calculated as body mass (kg) divided by height squared (m^2^). DXA will be used to assess, lean mass (kg), total body fat (%), bone mineral density (g/cm2), abdominal adiposity (g), truncal adiposity (g), and limb adiposity (g) (Lunar Prodigy, GE Healthcare, Madison, Wisconsin, USA; enCORE V.14.10.022).

### Blood pressure, vascular stiffness, and heart rate variability

Blood pressure will be assessed using a VaSera VS-2000 device (Fukuda Denshi, Japan). Participants will be asked to rest for 5 min before 5 blood pressure and heart rate readings will be taken at 2-min intervals by a trained study investigator. Pulse wave velocity (PWV) and cardio-ankle vascular index (CAVI) will be estimated by brachial-ankle oscillometry using a VaSera VS-2000 device (Fukuda Denshi, Japan) as markers of vascular stiffness according to Shirai et al. [45]. For each patient, a single determination will be used to determine the systolic and central and peripheral diastolic blood pressure variables, the CAVI index, and PWV with the participant resting supine for at least 10 min before the measurement. Electrocardiogram electrodes will be placed on both wrists, a microphone for detecting heart sounds will be placed on the sternum, and cuffs will be wrapped around both upper arms and above each ankle.

Heart rate variability (HRV) will be measured by the cardiofrequency meter Polar® Vantage V2™ (Polar Electro Oy, Kempele, Finland). This system captures the R wave of the ECG with a sampling frequency of 500 Hz, thus calculating the HR instantly. Low frequency (LF, 0.04–0.15 Hz), high frequency (HF, 0.15–0.4 Hz), and very low frequency (VLF, 0.0033–0.04 Hz) are different frequencies with varying power ranges. The ratio of LF to HF (LF/HF, %) is used to quantify the degree of sympathetic and parasympathetic balance in the body. In addition, the time domains measured in this study, including root mean square of successive RR interval differences (RMSSD) and the standard deviation of NN intervals (SDNN), will be reported as milliseconds (ms).

### Health-related quality of life and cognitive function and mental health

The Spanish version of the EuroQOL EQ-5D instrument [46] (concordance correlation coefficient: 0.948) will be used to assess health-related quality of life (HRQoL). The EQ-5D-5L was selected because it is an easy-to-use tool with adequate psychometric properties and can produce a single HRQoL index. In recent years, its use has been extended to general outpatient and in-hospital clinical research, which is consistent with the collection of HRQoL information for patients in the referral program [47]. In addition, the EuroQol visual analog scale will be used to ask patients to score their quality of life from 0 (worst imaginable health) to 100 (best imaginable health) before COVID-19 and at the time of the visit. *Cognitive function* will be measured by the Montreal Cognitive Assessment (MOCA) [48] (Cronbach’s *α* = 0.886) is a performance test that is used to screen for cognitive impairment. The score ranges from 0 to 30 points, with higher scores indicating better cognitive performance. A score ≤ 24 indicates cognitive impairment [49]. The Trail Making Test A [50] (reliability at least 0.76 for Part A) will be used to evaluate attention. For mood, participants will be evaluated via the brief Spanish version of the Community Epidemiological Study-Depression scale (CES-D), a 10-item self-report concerning the past week. The CES-D short form has shown high reliability and internal consistency [50] and validity for the identification of depressive symptoms [51]. Possible scores range from 0 to 30, with higher scores reflecting more severe depressive symptoms. To identify patients with probable clinical depression, we will use the recommended cutoff of 10 [51].

*Psychological distress* will be evaluated with the 10-item Kessler Psychological Distress scale (K10), which is a self-report instrument for the detection of nonspecific psychological distress. This scale has strong psychometric properties and is able to discriminate psychiatric cases from noncases [52, 53]. The K10 consists of 10 items with each five Likert-type response categories: “none of the time” (1), “a little of the time” (2), “some of the time” (3), “most of the time” (4), and “all of the time” (5). Sum scores range from 10 to 50, with higher scores indicating more symptoms or more frequent experience of those symptoms. *Resilience* will be measured by the Brief Resilient Coping Scale (BRCS), which is a 6-item measure designed to capture tendencies to cope with stress in a highly adaptive manner (internal consistency *α*=0.76, and test-retest reliability *r* = 0.71) [54]. Resilient coping is conceptualized as coping with stress in a highly adaptive manner, using a 5-point Likert scale “from ‘1’ = describes me not at all to ‘5’ = describes me very well.” Higher scores indicate that the individual perceives they have a better ability to “bounce back” and recover from stressful events and/or situations. *The chronic burden ongoing* (i.e., lasting for 7 weeks or longer) strains will be assessed in four domains: health of close others, work, finances, and relationships [55]. Respondents will be asked to indicate whether (yes, no) they are experiencing an ongoing problem in each of these domains and, if so, to indicate how stressful the ongoing problem is. We will sum the number of domains for which the respondent reports experiencing moderately stressful or very stressful ongoing problems (range, 0–4; 0 = no, 2 = yes, not very, 3 = yes, moderately, and 4 = yes, very stressful). We will also measure *life satisfaction* using one question: using a scale from 0 to 10 where 0 means “the worst possible life overall” and 10 means “the best possible life overall,” how would you rate your life overall these days? [56]. Last, *stress perception* will be measured by the 1 Item Statistics Canada Stress Question (SCSQ) with a possible score of 0 to 10, where higher scores indicate more stress.

### Questionnaire on sociodemographic and health-related data

The following data will be recorded in a standardized questionnaire by the staff researcher at baseline: sociodemographic data (age, sex, marital status, highest educational level, employment status, monthly income), risk factors (sleeping habits, eating habits, alcohol consumption, diet, physical activity levels), and health-related data (vaccines, self-reported diseases, medications, illness in family history).

### Safety group and assignment of possible events

The research team will consist of professionals from the health sciences and physical activity sciences, physical therapists, and rehabilitators with experience in cardiovascular management and safety in the clinical and primary care settings. Training, coaching, and standardization in the measurement of variables, programs, record sheets, and databases will take place. Additionally, the study will gather a group of specialists with experience in the management of exercise interventions (internal medicine) in accordance with the recommendations of the Tripartite Guide of the International Conference on Harmonized Good Clinical Practices to provide support and safety in case of possible adverse events. Study staff will collect data on spontaneously reported adverse events. Severe adverse events will be promptly reported to the Regional Ethics Committee of the HUN in accordance with the recommendations of the GBPC. Management of adverse effects will be based on participant protection and safety. The following adverse events will be recorded: signs of infections, hospital admissions, angina pectoris, and injuries/pain that prevent participation in training. Any serious adverse events (death, life-threatening event, hospital admission, expected or unexpected) will be reported to the Regional Ethics Committee of the HUN in accordance with the recommendations of the GBPC within 7 days after the study principal investigator or physician has become aware of the occurrence. These reports will include (a) recruitment and follow-up in terms of person-days and in comparison with the preestablished goals; (b) participant adherence (in terms of biochemical markers and data on the degree of compliance) and comparisons with the expected objectives of the exercise groups; (c) quality control (preferences for certain digits, variability, out-of-range values); and (d) intermediate and final variables and primary and secondary outcomes for each group and comparisons between groups with respect to the primary and secondary variables. A telephone number and email address will be made available to allow the monitors to make inquiries and resolve doubts. The program monitors will prepare a report on the participants’ attendance and, where appropriate, the possible reasons for drop out.

### Data collection and management

#### Plans for assessment and collection of outcomes

Study organization consists of the study steering committee (RR-V, MI, JMC- FT, YG-A, and JO), clinical center, biostatistics/methodology unit, and laboratory. The steering committee will finalize the protocol and will regularly communicate via telephone, email, and meetings. The biostatistics/methodology unit will generate randomized allocation and provide guidance on statistical analyses. Weekly investigator and clinic staff meetings will be held.

To assess the veracity of the questionnaire data and the database itself, a team of personnel external to each group will review the instrument and questionnaire responses of a random number of participants, approximately 50% of the participants in each group. All of the forms will be completed in duplicate, and subsequently, an exhaustive review will be carried out in search of missing data. The existence of inconsistencies will also be reviewed. To minimize potential errors, an operations manual has been written. Until the end of the trial, no trial information will be available to group personnel other than statisticians and members of the participant safety group. Only anonymized data will be recorded. All data will be stored on a secure server, and paper copies will be maintained in an appropriately locked and secured cabinet.

Adherence to training in both groups will be monitored by exercise trainers who will track attendance in training sessions and adherence to required training intensities as described in this protocol, using the RM% scale and Polar HR devices. If participants miss sessions of training, they will be offered make-up sessions to complete the full 12 sessions of training. During the period between T1 and T2, staff researcher will contact participants by telephone to encourage participant retention at T2.

### Statistical procedures

#### Sample size

Given the absence of exercise interventions for patients with persistent COVID-19 symptoms [57], in this area, a conservative approach will be used to estimate the sample size. Inspired by a Lau et al. study [58], it is expected that the exercise group will increase cardiorespiratory fitness at least 1-MET (3.5 mL/kg/min) more than the standard care group with an SD of 5.4%. A sample size of 84 participants (42 per group) provides statistical power (1−*β*) of 80% using a two-tailed alpha <0.05 and adjustment for baseline value and other prognostic covariates [59]. This sample size will also be sufficient for detecting differences in our secondary biomarkers and patient-reported outcomes. Considering a potential dropout rate of <15–20% based on our previous exercise trials [60, 61], a total of 100 participants (50 per group) will be randomized. This power will also be sufficient for detecting differences in our secondary biomarkers and patient-reported outcomes if the effects are moderate (i.e., effect size of Cohen’s *d* ≥ 0.6). This power is not sufficient for detecting potentially meaningful differences in any of the exploratory clinical outcomes. Given that the purpose of this phase II trial is to inform larger phase II and III trials, the patient-reported and clinical outcomes will also be interpreted for potential clinical significance based on the direction and magnitude of numerical differences. These calculations were performed with the Power and Sample Size Calculations program, Version 2.1.23.

#### Methods in analysis to handle protocol non-adherence and any statistical methods to handle missing data

The study follows an intention-to-treat principle with the use of baseline observation carried forward imputation, where the baseline value will replace missing data [61]. The final analysis population will be fully described and differences from the enrolled population will be presented. Cases that are lost may remain in the analysis, although they will provide less information and reduce the variance of estimated effects less than cases that are present for all measurement times. Adherence will be the number of group sessions attended/in sessions completed by each participant divided by the total number of sessions available × 100. Retention will be the number of participants who remained in the training program for all 6 weeks divided by the total number of participants × 100. Satisfactory adherence is defined as a minimum of ≥80% adherence to the prescribed RT sessions. Any discrepancies in data analysis between the methods will be resolved by a third-party biostatistician. First, descriptive statistics for the overall sample and between groups will be calculated. Second, a sequence 1–2 crossover analysis will be conducted using *t* tests and/or ANOVA to determine changes in primary and secondary continuous outcomes with individual participants as blocking factors. Categorical outcome measures will be analyzed using a McNemar test for data combined across both periods of the crossover. In addition, the proportions of participants reporting or not reporting long-term COVID-19 symptoms at the end of each period will be compared using a 2-proportion *z* test. If this test yields a statistically significant result, the usual test for differences between the effects of the two treatments should not be applied. By neglecting carryover effects using the procedure of Gaus and Högel [62], we expect enough power for investigation of differences. All analyses will be 2-sided. An *α* value ≤.05 will be considered to indicate statistical significance for the primary analysis.

#### Methods for additional analyses

Subgroup analyses will be conducted to understand if the exercise is more or less effective for PBMCs and metabolomic analysis than no-exercise group. These subgroup analyses will follow the same plan as the primary analysis will be included as well as its interaction with the experimental condition. All analyses will be performed using commercially available statistical software.

### Plans for communicating important protocol amendments to relevant parties

Protocol amendments will be approved of by the IRB prior to implementation. If relevant, current participants will be informed of protocol modifications. The ClinicalTrials.gov registry for this study will be updated with important protocol amendments.

### Plans to give access to the full protocol, participant-level data, and statistical code

The study is registered on ClinicalTrials.gov (NCT04797871). A complete, cleaned, de-identified copy of the final dataset used in conducting the final analyses will be made available within 1 year of study completion to protect the identity of interviewees. Outside investigators will be required to follow all the protocols for confidentiality, security, notifications to the study sponsor (Plan Estatal de Investigación Científica y Técnica y de Innovación 2017-2020), acknowledgments of funding, etc. required of the research team.

### Frequency and plans for auditing trial conduct

There are no plans for auditing trial conduct.

## Discussion

The present study will investigate the modification in immunological parameters, physical condition, inflammatory profile, and changes in perceived persistent symptoms (fatigue/tiredness, musculoskeletal pain, shortness of breath) after 6 weeks of multicomponent and supervised physical exercise in addition to the standard medical treatment scheme in patients with persistent COVID-19 symptoms. Recent experimental evidence has shown that skeletal muscle is capable of modifying low-grade subclinical inflammation and modulating the immune system and endocannabinoids. The EXER-COVID Crossover Study is a radical innovation that explores the possibility of reducing the degree of perception of persistent symptoms. Specifically, the primary purpose of this proposal is to implement precision exercise medicine that modulates the immune system, physical condition, and inflammation. The EXER-COVID Crossover Study is expected, based on the knowledge and cutting-edge developments provided by different researchers, to offer a nonpharmacological intervention that provides personalized management of the persistent symptoms of COVID-19. Since we will provide preliminary insights into the effect of a multicomponent exercise program on immunological parameters, physical condition, inflammatory profile, and persistent perceived symptoms, the results obtained should be of special interest for the design of health promotion and prevention programs for patients with persistent COVID-19 symptoms at the national and international levels. We expect that the information obtained by this study will inform future guidelines on the exercise training rehabilitation of patients with postdischarge symptoms after COVID-19.

Interestingly, by using a crossover trial, we minimize the risk of confounding because all interventions are measured on the same participants. One can say that study participants serve as their own control. This is achieved via computing the treatment effects separately in two sequence groups formed via randomization. Another advantage which is less study participants is required than parallel-group trials to meet the same criteria in terms of type I and type II error risks, when studying new interventions [63].

## Conclusions

As previously stated, we anticipate that the results obtained by this study will inform future guidelines on the exercise training rehabilitation of long-term COVID-19 patients.

## Trial status

Recruitment started in March 2021. Initially, recruitment of participants is expected to be completed in May 2022. However, due to the current pandemic, recruitment may be delayed. It is expected that recruitment will be completed by September 2022.

## Dissemination plans

The results of this study will be disseminated through publication of the findings at peer-reviewed literature, conference proceedings, blog posts, and policy briefs.

## Supplementary Information

**Additional file 1.** SPIRIT Checklist for Trials.

## Data Availability

We will publish the participant-level, de-identified data used in a publicly available online data repository within 1 year of study conclusion. Data used to analyze subgroup sample are available upon reasonable request to PIs RR-V and MI.

## Abbreviations

RT: Supervised resistance training
ACE-2: Angiotensin-converting enzyme receptor 2
BRCS: Brief Resilient Coping Scale
CAVI: Cardio-ankle vascular index
CES-D: Community Epidemiological Study-Depression scale
COVID-19: Coronavirus disease 2019
DXA: Whole-body dual-energy X-ray absorptiometry
HF: High frequency
HR: Heart rate
HRQoL: Health-related quality of life
HRV: Heart rate variability
IL: Interleukin
K10: 10-item Kessler Psychological Distress scale
LF/HF: Ratio of LF to HF
LF: Low frequency
MOCA: Montreal Cognitive Assessment
NK: Natural killer
PBMCs: Peripheral blood mononuclear cells
PROMs: Patient-reported outcome measures
PWV: Pulse wave velocity
RMR: Resting metabolic rate
SARS-CoV-2: Severe acute respiratory syndrome coronavirus 2
SCSQ: Statistics Canada Stress Question
TNF-α: Tumoral necrosis factor-alpha
UHPLC–MS: Ultrahigh-resolution liquid chromatography coupled to mass spectrometry
VLF: Very low frequency
V̇O_2_max: Maximal oxygen uptake
V̇O_2_peak: Peak oxygen uptake
1 RM: 1-repetition maximum
